# Arctic Sentinels: Global mercury emissions have stabilized over the past decade, yet levels in Arctic marine mammals have risen by an order of magnitude. Scientists struggle to explain why.

**DOI:** 10.1371/journal.pbio.0060259

**Published:** 2008-10-21

**Authors:** Hannah Hoag

## Abstract

Global mercury emissions have stabilized over the past decade, yet levels in Arctic marine mammals have risen by an order of magnitude. Scientists struggle to explain why.

On a cloud-covered morning in April, Feiyue Wang trudges down the gangway of the Canadian research icebreaker CCGS Amundsen and joins three graduate students and a rifle-toting research technician on the ice below. They set out on snowmobiles across the snow-covered slab of ice adrift in the Beaufort Sea and stop at an untouched patch of the ice floe. They unload coolers, thermoses, a generator, and a variety of electric tools, as the hems of their jackets whip about in the wind. It's −15 degrees Celsius, and the winds are gusting to 30 knots. The technician strolls along the edge of the field site with the rifle slung over her shoulder and scans the horizon for polar bears.

For most of the group, it's their first chance to be “on the ice” since they arrived. They've endured a full day of travel and another of safety drills and lab cleaning—Michel Gosselin, the chief scientist for this leg of the expedition, has made it clear that although their work is important, no science would be done until the labs are straightened up and bench top materials secured in case the ship had to suddenly dislodge from the ice floe. Samples of algae, zooplankton, water, and mud have piled up in the laboratories, which are passed on from one group to the next while the Amundsen is at sea.

Despite the harsh conditions, the team works for hours without a break. They extract four-foot-long (~1.25-meter-long) ice cores from the floe and scoop up surface snow and seawater (see Figure 1). They will later analyze the samples in the onboard laboratories (see Box 1) to study the presence of mercury and other contaminants, tracing their paths from terrestrial and atmospheric sources to ocean waters and, ultimately, to marine mammals. Wang, an environmental chemist from the University of Manitoba in Winnipeg, Canada, and his collaborators believe that climate change may be behind a recent jump in mercury accumulation in beluga whales, ringed seals, and other marine mammals.

**Figure 1:**
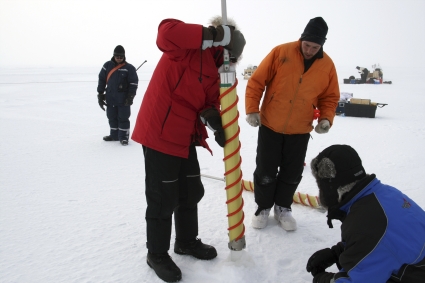
Getting to the Core of Climate Change When the Amundsen is parked in an ice flow, ice coring is a regular activity. Sometimes the ice team will spend the whole day outside to cut more than 100 ice cores. These ice-coring parties round up as many graduate students, postdocs, research assistants, and professors as possible to assist in the cutting and plucking of cores. Usually, a hollow cylinder called a core-barrel is attached to a drill or a gasoline-powered motor; but sometimes cores are cut by hand. When the team is done cutting cores, the ice floe resembles a slab of Swiss cheese. In the spring, a thin mat of orangey-brown sea ice algae covers the end of the core—at the sea–ice interface. The algae's abundance depends on the availability of nutrients in the water column and the amount of sunlight that penetrates the ice—and, therefore, the thickness of the ice and the depth of the snow. The layers of algae will be thicker where the snow and ice are thinner. A row of small glass vials filled with water and small mossy brown fragments in the onboard biology laboratory shows this: each vial contains more algae than the next. The samples were taken from ice cores extracted from areas of high snow depth to low snow depth. (Photograph by Hannah Hoag)

**Box 1: Keeping It Clean**

One of the unique features of the Amundsen is its onboard clean lab, built solely for the study of mercury. The lab is nestled in a white shipping container that has been chained to the aft deck of the ship. Few people are allowed access to the clean room, and no one gets though the inner door without removing her heavy down coat and boots, and donning a white zip-up suit, booties, and a cap. Inside, machines cover the bench tops and are secured by a complex system of bungee cords and hooks. Should the Amundsen begin to move or be jostled by high winds, the equipment needs to stay in place.

The clean room is stocked with equipment that can measure different mercury species in snow, ice, brine, and water samples in near–real time. “We used to ship the samples back to Winnipeg,” says Gary Stern, a researcher with the Department of Fisheries and Oceans and a professor at the University of Manitoba, “but that was always an issue. The possibility of contamination was high and you wouldn't know it until you got back.”

The Amundsen is the research base for Canada's largest research project of the International Polar Year, the Circumpolar Flaw Lead System Study—a Can$40 million study of the impact of climate change on the Arctic ecosystem (see Figure 2). The researchers on board are especially interested in flaw leads, or polynyas, which are long watery gaps in the ice that open up when the mobile sea ice pulls away from the stationary coastal ice. Flaw leads can stretch for many kilometers and extend throughout the Arctic region, like oblong beads on a necklace. They are frequently covered with thin new ice, or nilas, in the winter, and have high levels of primary productivity during the warmer months. They are particularly sensitive to environmental change. The Amundsen is the first icebreaker to spend the winter in or close to the flaw lead.

**Figure 2:**
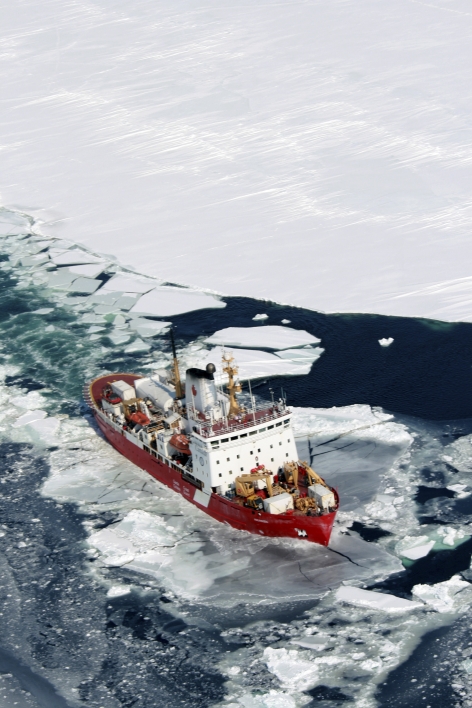
Canadian Coast Guard Ship Amundsen In 2003, a consortium of Canadian universities and research centers received Can$27.7 million from the Canada Foundation for Innovation to retrofit a decommissioned icebreaker, the CCGS *Sir John Franklin*, into a state-of-the-art arctic research vessel. The storage holds were transformed into laboratories, and a moon pool—an opening in the bottom of the ship that allows scientists to lower instruments and remotely operated vehicles (ROVs) into ice-covered waters—was added. (Photograph by Hannah Hoag)

Flaw leads are expected to become more prevalent in the years ahead, as rising temperatures and melting sea ice create an increasingly ice-free Arctic. Flaw leads may be longer and wider and covered with thinner ice in the winter and early spring. “They're a proxy of what can happen in the future,” says Gosselin. By studying the flaw leads, researchers hope to understand how climate change might alter mercury cycling in the Arctic and how both may interact to affect the Arctic ecosystem.

## Mercury Rising

There was a time when the Arctic was considered one of the last pristine spots on Earth. Over the last 30 years, scientists have detected heavy metals, persistent organic pollutants (POPs), and other chemicals in the air, seawater, snow, and wildlife in the Arctic. Coal burning, metal mining, waste incineration, and other industrial activities have been releasing mercury into the atmosphere since the industrial revolution. Elemental mercury is highly volatile, enabling its long-distance transfer from equatorial, subtropical, and temperate regions toward the poles. During the summer months, major air currents in the Northern Hemisphere lead to the Arctic.

Some mammals in the Canadian Arctic have shown 10-fold increases in mercury levels since the end of the 19th century. Of particular concern are the steep increases of mercury—a potent neurotoxin—in beluga whales and other marine mammals that have been hunted for food by northern peoples for centuries.

Gary Stern, a researcher with the Department of Fisheries and Oceans and a professor at the University of Manitoba, and his colleagues measured mercury levels in samples of liver, kidney, muscle, and muktuk—frozen whale skin and blubber, frequently eaten by the Inuit—taken from beluga whales from the Mackenzie Delta between 1981 and 2007. Stern says the highest mercury concentrations were measured in animals from the mid-1990s. The levels have since declined a bit, but they remain higher than they were in the 1980s [1], even though global atmospheric mercury has been in decline since the 1980s. And in the high Arctic, where scientists have been measuring atmospheric mercury levels since 1995, mercury levels have remained stable, and may be decreasing. “We didn't see an increase in mercury that could explain the mercury concentration in the biota,” says Wang.

Stern and other scientists are investigating the list of possible processes that might be driving the higher mercury accumulation in animals in the Beaufort Sea, but an explanation for this phenomenon has been elusive. Although biomagnification is an important factor in the accumulation of mercury in top predators, like beluga, Stern says that something else is increasing the bioavailability of the mercury already in the system.

One possibility is that increasing amounts of atmospheric mercury are being transferred to the water column. The mercury cycle in the Arctic is complex and far from being fully understood, but scientists have been piecing it together since the 1990s. Most of the year, mercury exists in the atmosphere in its gaseous elemental form. But when the sun rises in the Arctic in March, after many months of darkness, it undergoes a substantial transformation. The polar sunrise signals the end of winter, bringing light and heat to the dark and icy landscape. It also sets off a chain of photochemical reactions at the sea–ice interface that scavenge mercury from the atmosphere. In a matter of hours, the gaseous elemental mercury hovering over the Arctic is deposited on the snow, ice, and open water.

These so-called mercury depletion events (MDE) were discovered in the mid-1990s when a team of Canadian researchers investigating atmospheric mercury levels at a remote Arctic research station noticed that mercury levels dropped in tandem with ground-level ozone [2,3]. In the winter, atmospheric mercury levels are stable, averaging 1.5 to 1.7 ng/m^3^. But after the polar sunrise, the concentrations fluctuate wildly, dropping to less than 0.1 ng/m^3^ within a day [4]. “This is one of the most interesting times of the year for mercury people,” says Wang, as he presents his research to an international group of graduate students, postdoctoral researchers, and professors, and a handful of curious Coast Guard officers and crew one night in early May. The polar sunrise brings light to the Arctic, but it also releases a cascade of mercury from the atmosphere to the snow, ice, and water. Snow samples collected in January and February contained less than 10 ppt of mercury. “This week, it's 140 ppt,” he says.

The mercury showers are “an amazing series of events,” says Sandy Steffen, an atmospheric chemist at Environment Canada, a federal agency. These showers are due to a confluence of conditions: ultraviolet radiation from the newly risen sun and the release of inorganic bromine from the sea-ice surface. When an area of open seawater—such as a flaw lead—refreezes, tiny pores in the ice called brine channels wick salty seawater to the surface. “Like the veins in our arms, there are veins all through the ice,” says Amanda Chaulk, a graduate student at the University of Manitoba and part of the Contaminants Team on board the Amundsen. Exposed to the cold air, the brine forms delicate crystalline structures called frost flowers that are enriched in bromine, a chemical found in seawater. As the sunlight scatters across this garden of frost flowers, ultraviolet radiation collides with the salts and breaks down the bromine into radicals, which then react with ozone to form oxygen and bromine oxide radicals. These, in turn, oxidize the elemental mercury into reactive mercury (Hg^2+^), a chemically sticky substance that can bind to small particles and precipitate onto the snow and ice, or into the water.

Researchers suspect this phenomenon occurs only in areas of new sea ice, but they have yet to look into what happens around multiyear ice. “The Amundsen is such a unique platform for us. We get right in the middle of the action, to the middle of the open leads as the frost flowers are forming,” says Steffen, who was on board the Amundsen for five weeks this spring.

For some time, scientists thought MDEs might explain the increased mercury levels that they had observed in the Arctic marine biota. Now some aren't so sure. “We thought we had the smoking gun,” says Stern. “Now we see that it is a red herring.” Instead of staying on the snow and ice, some studies show that as much as half of the mercury deposited during an MDE can be photoreduced and returns to the atmosphere within four days of the event [5]. If this is correct, then MDEs could have less of an impact on polar ecosystems than previously anticipated.

“We've learned that there are a number of different potential drivers that could be behind the mercury concentrations we are seeing,” says Stern. Increased inputs from the Mackenzie River, melting permafrost, and the lack of thick, multiyear ice may bear some responsibility for adding mercury to the Arctic food chain.

Mercury is not readily absorbed by organisms in its inorganic form. But when it is converted to methylmercury (likely by cold- and mercury-resistant microbes), this highly toxic form can bioaccumulate in the food chain. Methylmercury is gobbled up by the phytoplankton that bloom in the water column and the sea ice algae that cling to the underbelly of the sea ice during the spring. These marine plants are swallowed up by zooplankton and fish larvae, which are, in turn, consumed by larger fish, seals, walrus, whales, polar bears, and people. Traditional northern diets—which contain large amounts of marine mammals and fish flesh, and in some cases, the kidneys and livers of fat-heavy seals, whales, and walrus—expose the northern people to mercury levels that are among the highest in the world. When Stern measured mercury levels in the liver, kidney, muscle, and muktuk of beluga, he found that nearly all the liver samples contained mercury at concentrations above the Canadian consumption guidelines for fish of 0.5 μg of total mercury per gram of fish tissue [1]. (There are no consumption guidelines for marine mammals.)

During the 1970s, researchers began to detect high levels of methylmercury in the blood and hair of many of the indigenous residents [6]. In June, Eric Dewailly, a public health specialist at Laval University, in Quebec City, reported that 28% of the general population in Nunavik had blood mercury concentrations higher than the acceptable blood concentration established by Health Canada (99.7 nmol/l for the general population; 28.9 nmol/l for women of childbearing age) [7]. Researchers have documented blood mercury levels as high as 660 μg/l among people living in the Canadian north (compared with 2 μg/l in populations that consume little or no fish), as reported in a 1999 review of human health impacts of Arctic environmental contaminants [8]. (1 μg/l is approximately equal to 5 nmol/l.)

In adults, methylmercury accumulates in the brain and damages the central nervous system. The effects of prenatal exposure have been inconclusive, however. While some studies have found that infants exposed to methylmercury in utero showed developmental delays in cognitive and sensory functions, others have not. In Nunavik, Dewailly and his colleagues have found that prenatal mercury exposure correlates with subtle neurodevelopmental consequences, leading to slight hand tremors, for example, and difficulties in processing visual information [9,10] in pre-school–aged children.

## An Ice-Free Future?

Some researchers, including Stern, think that climate change may be behind the recent rise in marine mammal mercury levels. The expansion and contraction of the floating Arctic ice cap is one of the Northern Hemisphere's natural cycles. Normally, sea ice decreases during the Arctic summer and recovers when temperatures drop in the winter. From 1978 to 2000, the average sea ice extent, the area of ocean covered by at least 15% of ice, hovered around 7 million km^2^ during the summer minimum. But since 2002, there have been sharp drops in the region's minimum ice coverage. In September 2007, a mere 4.3 million km^2^ of ice covered the area. Some scientists have projected that the Arctic might see ice-free summers as early as 2013.

In March, researchers on the Amundsen were unable to set up a science station on a fast ice bridge that normally stretches between Cape Parry on the mainland and Nelson Head on the southern tip of Banks Island, because the winter ice had formed quite late and wasn't thick enough. This year's flimsy ice is the result of last year's extensive melt. By the end of August, the Arctic sea ice extent was on the verge of another all-time low, and the direct route through the Northwest Passage was almost ice free. “Not having that ice is like removing the skin off the surface of the water,” says Stern. “If you remove the trees from the rain forest, it will affect a whole range of things. It is the same for the ice and the water.”

The warmer temperatures could have unforeseen effects on atmospheric contributions to mercury levels in the Arctic. Depletion events depend heavily on areas of new sea ice. Initially, Steffen says, reduced sea ice cover could lead to larger open leads and new sea ice and create better conditions for MDEs that could add more mercury from the atmosphere to the aquatic system. On the other hand, warmer temperatures will melt more snow and can shut down the depletion event. “Until we totally understand the processes, we're not going to understand the effects,” says Steffen.

## Exploring a Role for Arctic Warming

Until recently, very little attention had been paid to the non-atmospheric mercury transport pathways into the Arctic, including ocean currents, rivers, and coastal erosion. Compared to the atmosphere, the ocean in the Arctic is a bit of a black box, says Peter Outridge, an adjunct professor at the Centre for Earth Observation Science, University of Manitoba. As rising temperatures cause sea ice, snow, and permafrost to melt, they are releasing once–locked-up mercury into the marine ecosystem. Runoff from the Mackenzie River and coastal erosion are draining naturally occurring mercury and carbon into estuaries and the edges of the Beaufort Sea, where the warmer water conditions hasten the conversion to methylmercury.

In April, Outridge and his colleagues published the first mass balance study of mercury in the Arctic Ocean [11]. Using the best available data for the inflows, outflows, and total masses of mercury in the atmosphere, rivers, ocean, sea ice, and sediments, along with mercury levels in bacteria, phytoplankton, zooplankton, fish, seals, whales, and polar bears, the researchers estimate that there are more than 47 metric tons of abiotic methylmercury in the upper ocean and 450 metric tons in the entire Arctic Ocean, but only 4.5 metric tons of methylmercury in the marine biota. “There is all this unassimilated methylmercury hanging out in the upper ocean,” says Outridge. “Even if you were to increase atmospheric deposition over the next 20 years, it won't change things much.” With so much mercury in the ocean already, it seems unlikely that the recent rise in mammals could be due to additional inputs from the atmosphere.

Instead, they speculate that sea ice plays a dominant role in the transfer of mercury to the biota. Sea-ice cover is one of the key factors affecting the primary productivity of the Arctic Ocean. In flaw leads, where the sea ice is thinner, sunlight penetrates more easily and promotes the spring growth of under ice algae, which gorge on the available nutrients, including those laced with methylmercury. Beluga and other marine mammals prefer to feed in areas of high productivity. “With beluga whales, the ice dictates where they feed,” says Stern. Rising temperatures—and the loss of sea ice—may have increased the whales' access to these high-productivity areas and their exposure to methylmercury.

If climate change is to blame for the mercury spikes in marine mammals, what can be done to reverse the trend? Mercury's long lifetime means that it will take several decades to reduce atmospheric mercury levels, much the same way that the effects of lowering carbon dioxide emissions may not be seen for years. Although reductions in atmospheric mercury will be necessary to return the mercury levels of arctic biota to normal, the response will be slow, says Outridge. But reducing greenhouse gases could be as important. “If it is climate change that is driving the increases, it will take hundreds of years to get back to where we were, but if you can stop reducing the impacts of climate change then you can theoretically go back to normal,” says Stern.

## References

[pbio-0060259-b001] Lockhart WA, Stern GA, Wagemann R, Hunt RV, Metner DA (2005). Concentrations of mercury in tissues of beluga whales (Delphinapterus leucas) from several communities in the Canadian Arctic from 1981 to 2002. Sci Total Environ.

[pbio-0060259-b002] Barrie LA, Bottenheim JW, Schnell GC, Crutzen PJ, Rasmussen RA (1988). Ozone destruction and photochemical reactions at polar sunrise in the lower Arctic atmosphere. Nature.

[pbio-0060259-b003] Schroeder WH, Anlauf KG, Barrie LA, Lu JY, Steffen A (1998). Arctic springtime depletion of mercury. Nature.

[pbio-0060259-b004] Steffen A, Douglas T, Amyot M, Ariya P, Aspmo K (2008). A synthesis of atmospheric mercury depletion event chemistry in the atmosphere and snow. Atmos Chem Phys.

[pbio-0060259-b005] Kirk JL, St. Louis VL, Sharp M (2006). Rapid reduction and reemission of mercury deposited into snowpacks during the atmospheric mercury depletion events at Churchill, Manitoba. Environ Sci Technol.

[pbio-0060259-b006] Kinloch D, Kuhnlein H, Muir DCG (1992). Inuit foods and diet: a preliminary assessment of benefits and risks. Sci Total Environ.

[pbio-0060259-b007] Fontaine J, Dewailly E, Benedetti J-L, Pereg D, Ayotte P (2008). Reevaluation of blood mercury, lead and cadmium concentrations in the Inuit population of Nunavik (Québec): a cross-sectional study. Environ Health.

[pbio-0060259-b008] Van Oostdam J, Gilman UA, Dewailly E, Usher P, Wheatley B (1999). Human health implications of environmental contaminants in Arctic Canada: a review. Sci Total Environ.

[pbio-0060259-b009] Saint-Amour D, Roy MS, Bastien C, Ayotte P, Dewailly E (2006). Alterations of visual evoked potentials in preschool Inuit children exposed to methylmercury and polychlorinated biphenyls from a marine diet. Neurotoxicology.

[pbio-0060259-b010] Despres C, Beuter A, Richer F, Poitras K, Veilleux A (2005). Neuromotor functions in Inuit preschool children exposed to Pb, PCBs, and Hg. Neurotoxicol Teratol.

[pbio-0060259-b011] Outridge PM, Macdonald RW, Wang F, Stern GA, Dastoor AP (2008). A mass balance inventory of mercury in the Arctic Ocean. Environ Chem.

